# Unsolved challenges of clinical whole-exome sequencing: a systematic literature review of end-users’ views

**DOI:** 10.1186/s12920-016-0213-6

**Published:** 2016-08-11

**Authors:** Gabrielle Bertier, Martin Hétu, Yann Joly

**Affiliations:** 1 Center of Genomics and Policy, McGill University, 740 Dr. Penfield Avenue, Montreal, Quebec H3A 0G1 Canada; 2 UMR 1027, Inserm, University of Toulouse III - Paul Sabatier, 37 allées Jules Guesde, F-31000 Toulouse, France

## Abstract

**Background:**

Whole-exome sequencing (WES) consists in the capture, sequencing and analysis of all exons in the human genome. Originally developed in the research context, this technology is now increasingly used clinically to inform patient care. The implementation of WES into healthcare poses significant organizational, regulatory, and ethical hurdles, which are widely discussed in the literature.

**Methods:**

In order to inform future policy decisions on the integration of WES into standard clinical practice, we performed a systematic literature review to identify the most important challenges directly reported by technology users.

**Results:**

Out of 2094 articles, we selected and analyzed 147 which reported a total of 23 different challenges linked to the production, analysis, reporting and sharing of patients’ WES data. Interpretation of variants of unknown significance, incidental findings, and the cost and reimbursement of WES-based tests were the most reported challenges across all articles.

**Conclusions:**

WES is already used in the clinical setting, and may soon be considered the standard of care for specific medical conditions. Yet, technology users are calling for certain standards and guidelines to be published before this technology replaces more focused approaches such as gene panels sequencing. In addition, a number of infrastructural adjustments will have to be made for clinics to store, process and analyze the amounts of data produced by WES.

**Electronic supplementary material:**

The online version of this article (doi:10.1186/s12920-016-0213-6) contains supplementary material, which is available to authorized users.

## Background

Whole-exome sequencing (WES) consists in the capture, sequencing and analysis of all exons of all protein coding genes in the human genome. Instead of analyzing the whole genome, composed of roughly 3 billion base-pairs, WES focuses only on the approximately 30 million base-pairs which are translated into functional proteins, in which mutations are the most likely to have a severe direct phenotypic consequence. WES can therefore be considered a much less costly and more efficient method of identifying all possible mutations in genes, compared to other methods such as genome-wide association studies or whole-genome sequencing (WGS) [1]. WES was originally used mainly to identify rare mutations contributing to Mendelian diseases, as compared with the many variants involved in common complex diseases [2], although this distinction can be considered artificial [3]. Methodologies evolve rapidly, and new software enable this technology to better detect complex genetic changes such as structural variants [4] and copy-number variants [5–7]. The integration of WES into healthcare is already underway, contributing to the development of personalized medicine [2]. It is currently used clinically for numerous purposes, ranging from diagnosis to disease prognosis and treatment decisions [8]. Analysing a patient’s exome through one test is now less costly than testing a number of specific genes, especially when little is known about the genetic background of the disease, although this analysis does “add layers of complexity to test interpretation” [9]. In addition to the technical challenges of making the technology fit for clinical diagnostics (improving exon capture, sequencing coverage, read length, accurate detection of insertion-deletions, and reduction of false positive and false negative rates), numerous hurdles have to be overcome to use WES in routine healthcare. A number of ethical, legal, social and policy challenges have been extensively discussed in the literature by scientific researchers as well as policymakers and professional societies [10–15]. Guidelines have been produced to respond to some of these challenges, notably that of reporting incidental findings (IF). The American College of Medical Genetics and Genomics (ACMG) published a policy recommendation on this topic in 2013 [16], which has been heavily discussed [17–20], and updated in 2014 [21]. The European Society of Human Genetics, in turn, published a recommendation in 2015 [22]. The Canadian College of Medical Geneticists published a position statement in 2015 to frame the “clinical application of genome-wide sequencing for monogenic diseases in Canada” [23]. But in order to design efficient policies aimed at enabling the responsible integration of WES into healthcare, there is the need to systematically identify what the prominent challenges are. To our knowledge no study has yet been published on the implementation hurdles identified directly by scientific researchers and medical doctors (technology users) reporting on the clinical use of WES. With this objective in mind, we designed a systematic review of the literature to identify the most important challenges directly reported by technology users.

## Methods

Our systematic literature review methodology was adapted from the PRISMA guidelines [24] and the Petticrew and Roberts practical guide [25]. The completed PRISMA flow diagram and PRISMA checklist, as well as the full articles dataset are available in Additional files 1, 2 and 3.

### Studies sources

6 databases were searched to identify the most comprehensive list of publications. The last search was performed on March 31^st^, 2015.

1. EBSCO host digital archives http://www.ebscohost.com/archives
2. Embase http://www.elsevier.com/online-tools/embase/
3. NCBI Pubmed http://www.ncbi.nlm.nih.gov/pubmed/
4. Science Direct http://www.sciencedirect.com/
5. Scopus http://www.scopus.com/
6. Web of Science http://apps.webofknowledge.com/

### Choice of keywords

Since our objective was to identify reports published by technology users on the clinical use of WES, we used the following keywords in combination to stringently filter out reports from outside the clinical context: Clinical application, Medical application, Healthcare, Clinical care, Medical care, Clinical practice, Clinical diagnostic, Medical practice.

Therefore, the complete search used was the following:

(“exome sequencing” OR “whole-exome sequencing” OR “whole exome sequencing”) AND (“clinical application” OR “medical application” OR “healthcare” OR “clinical care” OR “medical care” OR “clinical practice” OR “clinical diagnostic” OR “medical practice”).

### Screening, filtering and selection

We searched for the chosen keywords using the full text of articles and reports without any date or language restrictions. The search resulted in 2275 articles (details available in Table 1). All results were then aggregated in a single Excel file, from which we removed duplicates, resulting in 2094 unique articles.

**Table 1:** Total number of hits by database

Database searched	Total hits
EBSCO academic search complete	893
EMBASE	258
NCBI Pubmed	123
Science Direct	722
Scopus	160
Web of Science	119
TOTAL	2275
Total unique articles	2094

Further screening was done in two steps:

- First, we screened out results that were not peer reviewed journal articles (such as abstracts from conference oral presentations or posters, blog articles, or conference programs).
- We then removed articles that were not written in English, French or Spanish.

At this point, both GB and MH processed with filtering the articles in parallel, according to the following inclusion and exclusion criteria:

- The articles are written by a technology user, defined as a medical doctor, life science researcher or medical researcher who is directly exposed to the technology in his field of expertise. At this point we excluded articles for which the corresponding author was a researcher in policy or human and social sciences.
- The articles directly address WES. At this point we excluded articles which, for instance, simply referred to other studies which had used WES. We included articles that talked about other technologies in addition to WES, such as WGS or gene panels.
- The articles discuss the clinical implementation of WES. At this point we excluded articles which only considered WES in the context of basic research or discovery.
- The articles list unsolved implementation challenges. We excluded articles which did not mention any challenge linked to the clinical implementation of WES, which listed challenges already solved, or which described them as easy to solve through measures already partly in place. We also excluded articles which we tagged as ‘recommendations’ when they consisted of a list of solutions for the clinical implementation of WES and did not describe any challenge or issue as ‘unsolved’.

After filtering all articles separately, GB and MH compared their selected articles list, discussed any articles selected only by one of them, and agreed on a final decision for each of those articles. Only 10 % of articles required discussion (182 out of 1792 articles).

The full list of selected articles is available in Additional file 1. The results of all screening and filtering steps are described in Table 2.

**Table 2:** Screening and filtering process

	Total	Removed
Total Articles	2275	
Screening		
Removing duplicates	2094	181
Peer reviewed journal articles	1810	284
Written in English, French or Spanish	1805	5
Accessible	1792	13
Filtering		
Included	147	1645

### Coding

Since our objective was to be as comprehensive and unbiased as possible in the identification of unsolved challenges relevant to technology users, the coding of articles was done through inductive content analysis [26, 27]. An initial list of challenges was generated by GB on the basis of an analysis of 30 articles selected at random (20 % of all selected articles). These challenges were then discussed and adjusted by all co-authors together. Some similar challenges were merged, while others were split into separate challenges. Additional challenges were added both by MH and GB over the course of the analysis if five articles or more were found to refer to any specific challenge. For the data analysis, we decided to group challenges along a typical ‘timeline’ ranging from data production, to analysis, reporting and finally sharing.

## Results

### Studies scope

#### Publication dates

The first articles selected were published in 2010, which is consistent with the appearance of WES technology in the scientific literature. 3 articles (2 %) were published in 2010, 13 (9 %) in 2011, 31 (21 %) in 2012, 42 (29 %) in 2013, and 46 (31 %) in 2014 and 12 (8 %) in the first trimester of 2015, when we performed the search.

#### Whole-exome sequencing/Next-generation sequencing/High-throughput sequencing

Among the selected articles, only 48 (34 %) focused exclusively on WES. The other 94 articles either discussed challenges linked to other technologies such as WGS or large gene panels, or discussed challenges linked to Next-Generation Sequencing or High Throughput Sequencing (including WES and other technologies) in general.

#### Article types

**Fig. 1:**
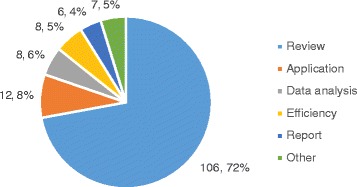
Selected articles types. Review: the authors do not report directly on the way they personally use WES, but rather review the current body of evidence about a certain aspect of the technology. Application: authors report on the application of WES on a specific patient, family, or a larger group of patients in a healthcare service. Data analysis: authors focus on challenges linked with WES data processing, analysis and interpretation. Efficiency: authors compare the efficiency of WES compared to other techniques, such as gene or gene panels sequencing. Report: authors report on the use of a technology other than WES, and explain this choice by identifying challenges with WES

A graph representing all article types is available in Fig. 1. Of the selected 147 articles, the vast majority (106, 72 %) are review articles in which the authors do not report directly on the way they personally use WES, but rather review the current body of evidence about a certain aspect of the technology. The majority of review articles (66, 62 % of reviews) describe the impact of WES on a specific disease or disease group, and 5 (5 % of reviews) generally discuss its use in the diagnosis of various diseases, whereas 25 (23 % of reviews) review the technology in general, including both its research and clinical applications. 6 articles (6 % of reviews) describe how the technology may impact a specific medical field, such as nursing [28] or pathology [29] while 4 (4 % of reviews) focus on pharmacogenomic applications. 12 articles (8,2 %) report directly on applications of the technology for a specific patient [30, 31], a family [32], a selected group of patients [33–35], or on a larger scale for a particular healthcare service [36–41]. 8 articles (5.4 %) discuss the efficiency of WES compared to other techniques, such as gene panels or WGS. 6 articles (4 %) report on the use of a technology other than WES, and explain this choice by identifying challenges with WES. Finally, 8 articles (5,4 %) focus on challenges linked with WES data processing, analysis and interpretation.

#### Disease focus

Our first observation was that the articles selected cover an extremely wide range of diseases, from cancer (26, 29 %) to rare diseases (24, 16.3 %) to common disorders such as intellectual disability and developmental delay (6, 4 %). 14 (9.5 %) articles focus on a diversity of heart diseases, 13 (14 %) on neurological diseases, and 3 (2 %) respectively on blood, muscle, and kidney disorders. It is a particularly challenging task to group the diseases addressed by our selection of articles in relevant categories for three main reasons. Firstly, those categories may partly overlap: for instance, cancer in children is considered to be a rare disease. Secondly, a number of articles (9, 6 %) focus generally on genetic or inherited disorders, which may or may not be rare diseases. Thirdly, some articles cover many possible diseases – such as cancer [42, 43] or rare diseases [44, 45] in general - while others focus specifically on one disease [46–48]. A significant number of articles (42, 29 %) did not focus on any diseases in particular, but addressed the impact of WES on all clinical contexts.

#### Country

**Fig. 2:**
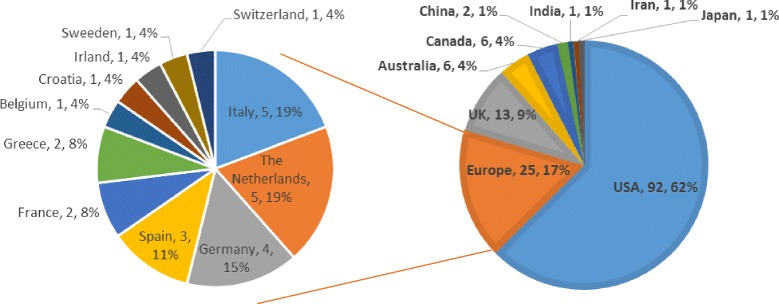
Number of articles per country of institution of corresponding author

We noted the country of the institution of corresponding authors of all selected articles. A total of 19 countries were represented. The majority of articles (92, 62 %) we selected were written in the USA. 25 (17 %) were written in Continental Europe (excluding the UK, which represented an additional 13 articles). The complete distribution of articles per country is represented in Fig. 2.

#### Number of challenges covered

**Fig. 3:**
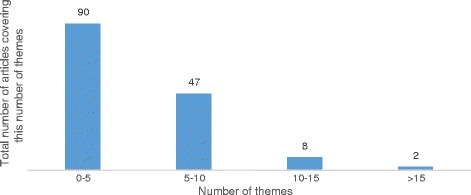
Number of challenges covered across articles

On average, the 147 selected articles covered 8 of the identified challenges. The majority of articles (90, 61.2 %) covered from 1 to 5 challenges. 47 articles (32 %) covered between 6 and 10 challenges, and only 10 articles (6.8 %) covered more than 10 challenges. This steadily decreasing distribution shows the importance of the systematic review methodology in identifying all challenges linked to the clinical implementation of WES as identified by technology users. This distribution is displayed in Fig. 3.

### Unsolved challenges identified

From the original 147 studies, we identified 23 unsolved challenges. These were divided into 4 categories, following the ‘samples and data trajectory’, of production, analysis, reporting and sharing.

**Fig. 4:**
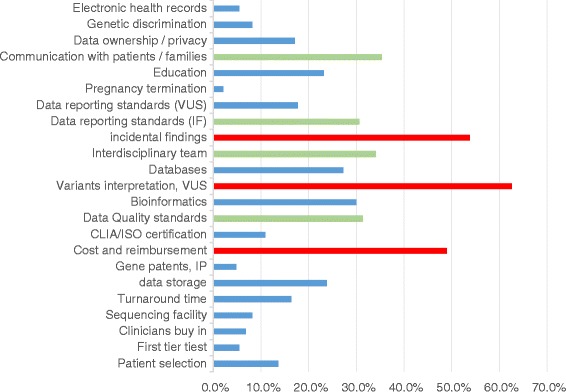
List of unsolved challenges and proportion of articles reporting on them. We highlighted the challenges found in more than 40 % articles (58 total) in *red*, and challenges found in 30 to 40 % articles (44 to 58) in *green*

Table 3 briefly describes the challenges found in all articles. Figure 4 displays the total number of articles covering each challenge. The unsolved challenges reported by technology users are extremely diverse, ranging from very specific challenges, such as the inclusion of WES results in patients electronic health records, to much broader ones, such as the challenges of communicating results with patients and their families and managing their expectations. Three challenges (henceforth referred to as major challenges) were reported by more than 70 (47,6 %) articles:

- the interpretation of variants and variants of unknown significance (VUS) was reported by 92 (62.6 %) articles
- challenges linked to incidental findings were reported by 79 (53.7 %) articles
- the cost of WES and reimbursement of the test by the healthcare system was reported by 72 (49 %) articles.

**Table 3:** Description of challenges identified

Category	Challenge	Description
Data production	Patient selection	It is difficult to determine which patients would receive a clear clinical advantage from WES.
	First tier test	It may not be clear whether WES should be used as a first tier test, or as a second tier test after the failure of more selective genetic testing such as gene(s) or gene panel(s) testing.
	Clinicians buy-in	Some clinicians are not willing to order WES testing, sometimes because of lack of trust in the technique. This can be an important barrier to clinical implementation of WES.
	Sequencing facility	Decisions will have to be made about whether sequencing should be done in each laboratory offering the test, or if laboratories should order it from centralized sequencing facilities.
	Turnaround time	WES results can sometimes take longer to obtain than more targeted tests, which may challenge their implementation in a clinically relevant timeframe.
	Data storage	WES data requires a large and secure storage space, which may not always be available in a clinical setting.
	Gene patents	In some jurisdictions, patents on the sequence of specific genes may make it difficult to sequence whole exomes without having to pay IP rights.
	Cost and reimbursement	The cost of WES sequencing and analysis may be too high for some clinical applications. Reimbursement strategies for such tests are yet to be established by private insurers and by the healthcare systems.
	CLIA/ISO certification	WES has yet to be standardized in order to obtain CLIA and ISO certification, in the USA and in Europe respectively. This certification is key for clinical implementation and reimbursement of WES by the healthcare systems.
	Data quality standards	There is still no formal agreement on the appropriate quality standards to apply to the technology so that it can be implemented in the clinic.
Data analysis	Bioinformatics	Analysis of WES results relies on a number of bioinformatics tools that have yet to be perfected.
	Variant interpretation, VUS	WES generates a high number of variants per individual, a large proportion of which are still of unknown significance. The extreme difficulty of interpreting these variants has created a bottleneck in the clinical application of the technology.
	Databases	To better interpret variants, WES and more generally NGS results need to be broadly shared. More complete and reliable reference databases linking variants to patients’ phenotypes need to be developed.
	Interdisciplinary team	The interpretation of variants relies on the collaboration of different professionals, including medical doctors, bioinformaticians, biologists and clinical geneticists. Integration of WES into the clinic may require that we reconsider the definition of new and established professional roles in clinical hospitals.
	Incidental findings	WES has the potential to generate a high number of incidental findings. These may create anxiety in patients and the need for costly follow-up procedures if reported.
Reporting	Data reporting standards (IF)	There is a pressing need to develop standards on which a large part of the community can agree regarding whether and how to report IF to patients and their families.
	Data reporting standards (VUS)	There is a pressing need to develop consensus standards on when and how to report VUS to patients and their families.
	Pregnancy termination	WES may enable the detection of mutations at a time when pregnancy termination is still possible, which was not possible with prior technologies. This leads to the necessity to develop new policy decisions which take into account the ethical justifications behind offering pregnancy termination options for these conditions.
	Education	Increased use of WES in the clinic will mean that a growing number of healthcare professionals will need to interpret these data, and therefore need to be educated in the basics of genetics and genomics. This is not the case today, as very few medical staff currently have genomics knowledge.
	Communication with patients and families	The amount and complexity of the data produced by WES complicates the task of healthcare professionals who have to report WES results to patients. In specific circumstances, they may also have a duty towards some of their patients’ family members. Many more types of results will have to be explained, in longer and therefore more costly pre and post-test counselling sessions.
Sharing	Data ownership/privacy	Given that WES data is inherently identifying and provides some information on the present and future health status of the proband and their families, several privacy and ownership questions have to be resolved: Who owns WES data? How should the access and sharing of this data be regulated?
	Genetic discrimination	The possibility for insurers or insurance companies to access WES data may lead to greater discrimination against potential clients or employees based on their genetic background.
	Electronic health records	The correct interpretation of WES data often relies on accessing a complete description of patients’ phenotypic characteristics, which would be greatly facilitated by consulting electronic health records. However, before this can be done public health systems and hospitals will have to decide whether WES results should be added to patients’ electronic health records.

The following sections provide an overview of the terms in which these three challenges are described in the selected articles.

### Data analysis challenge: variants of unknown/uncertain significance (VUS)

The most important challenge mentioned by the selected articles was that of the lack of standards and the complexity of variants interpretation, along with the high risk of finding VUS, which Sutton et al., 2012 consider a ‘plague’ to the field of clinical WES [49]. Unlike targeted single gene or gene panel sequencing assays, WES usually generates a long list of mutations, a large number of which have no known significance. VUS are reported to represent the majority of variants identified by next-generation sequencing (NGS) technologies such as WES [38, 50], although much fewer VUS are found in WES than in WGS [51]. It is unsurprising that VUS is the most consistently reported challenge, as it lies at the heart of a network of connected challenges. The assessment of VUS' pathogenicity is a long, complex and expensive research process [52], which requires the collaborative intervention of different highly trained specialists [53] including bioinformaticians, biologists and clinicians [54]. This need for interdisciplinary collaboration, along with the way WES testing may challenge existing professional roles in the clinic, was reported as a challenge by 50 (34 %) articles. To interpret variants, these specialists rely on bioinformatics analysis pipelines made of imperfect algorithms [36, 37, 42, 55], referring to imperfect databases [44, 50, 56, 57]. The need to develop more efficient and standardised bioinformatics tools to filter, analyze and interpret WES variants was reported as a challenge by 44 (29.29 %) articles. The need to share NGS results and to develop more complete, less biased databases containing fewer false positive and false negative variant-phenotype associations was identified as a challenge in 40 (27.2 %) articles. As described by Jongbloed et al., [58], the “only reasonable way to deal with [the ascertainment of VUS] is to pursue maximum data dissemination in the scientific community”, who could accelerate the analysis of VUS by creating and sharing access to large scale databases gathering sequencing results from as many studies as possible. Certainly, the more sequencing results are shared, the less likely it is that variants identified in patients will never have been reported before. This vision is also shared by Xue et al. [50], who assert that “With more individuals from different ethnic groups sequenced through NGS, more rare variants will inevitably be revealed”, and by Lin et al. [59]: “Sifting through the millions of variants in an individual’s genome for the pathogenic mutation seems to be the most urgent task at hand. The creation of dedicated databases specifically for the purpose of clinical interpretation based on NGS results from a large number of normal controls and diagnosed patients will significantly help this endeavor”. Considering the current uncertainty involved in interpreting VUS, they can represent a heavy burden [60] if reported to a patient’s genetic counsellor or physician. Having access to this information may force clinicians to make a ‘judgment call’ [61] in trying to interpret VUS, and potentially report them to patients, which risks causing them unnecessary anxiety [62]. This dilemma is particularly prevalent in screening for mutations contributing to the genetic background of rare diseases. Indeed, some genes are only found to be mutated in 1 or 2 families in the world. It is therefore very difficult to estimate their pathogenicity and their exact impact on patients, which also makes genetic counselling significantly more challenging [63]. According to Rabbani et al. [64], this should be carefully addressed in the consent form, and discussed during the consent process. In Need et al. [35], the decision was taken at the onset of the study to not report any variants of ‘uncertain significance’ to the patients, regardless of whether or not they were later proven to have significance. In comparison, Ream et al. [65] performed a pilot study in which the need to explain VUS to the families of 6 pediatric drug-resistant epilepsy patients represented a significant challenge, which led them to conclude that “WES may raise more questions than it answers for some patients”.

### Incidental findings (IF)

The challenge of IF was also consistently mentioned in 92 (53.7 %) of selected articles. IF can be defined as information of clinical relevance which is found during the WES data analysis and which is beyond the scope of the original clinical condition for which the patient was ‘prescribed’ a WES test. According to Sankaran et al. [57], the “identification of actionable, IF during genome-wide DNA sequencing genetic studies is a major concern of many patients, as well as health care providers”, and this can “cause ethical and clinical dilemmas” [66]. The topic of genomic IF is heavily discussed in the literature, and two recently published reviews [67, 68] provide strong evidence showing that there is a lack of consensus on how to define, analyze, and report such variants to patients and research participants. Within our selection of articles, for instance, Lyon et al. [69] consider the term “IF” to be “misleading”. They prefer using the term “secondary findings”, which they argue better represents their importance and could help correct the view that such findings do not require significant time and effort to be analyzed, interpreted, and reported. In 2013, Sankaran et al. [57] stated that there was no consensus on just how frequently they are actually found in NGS data. However, several authors provide different estimates: in 2014, Xue et al. [50] provided references to support the claim that “the rate of reportable IFs can range from 1 to 8.8 %”, while Gecz et al. [70] argued that they range from 1 to 2 % of patients. Regardless of how often IF are found in practice, they have to be addressed in the patient pre-test counselling process [51, 71], and this ‘intensive genetic counseling’ [28] can be a “main issue” in practice [40, 72]. Incidental findings are viewed as a potential “additional burden” [65] and source of anxiety for patients and their families [62, 73, 74].

19 out of the 55 articles published since 2013 which mention IF as a challenge (34.5 %) refer to the American College of Medical Genetics and Genomics (ACMG) recommendation on reporting IF [16, 21]. This recommendation, which provides a list of 56 genes to be systematically searched for ‘actionable variants’, has clearly raised “concerns” [53] and debate on this topic rather than helping to resolve it. Even after mentioning these recommendations, articles published in 2014 refer to the reporting of IF by clinicians as “currently a subject of intense debate” [52], “one of the current, contentious debates” [73], or state that “there remains strong debate” [56] and an “ongoing discussion on how to best proceed with incidental findings” [75]. Malhotra et al. [42] specifically mention that “the methods of providing [incidental findings] to patients are not entirely clear, although some recommendations have recently been made by the American College of Medical Genetics and Genomics”. Even in 2015, this is still considered to be a “current debate” by Goldberg et al. [76], and Bender state that the discussion of this topic “will undoubtedly continue” [77]. This uncertainty on how to define and report IF is described as the justification for using targeted testing over WES and more generally NGS in certain clinical contexts, such as hematology [46] or for heart diseases [78]. The challenge of IF even leads Lohman et al. [75] to refer to WES and WGS as a”curse” as well as a”blessing”.

**Fig. 5:**
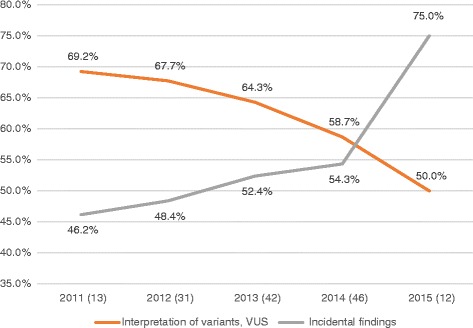
Proportion of articles addressing VUS and IF per year. In parenthesis next to the year of publication of articles, we indicated the total number of selected articles published that year

From 2011 to 2015, we noted a steady increase in the proportion of selected articles discussing the challenge of IF. Indeed, it rose from 46.2 % of articles published in 2011, to 75 % in 2015. This trend is opposed to that of the proportion of articles discussing VUS, as displayed in Fig. 5. Since the total number of selected articles published per year is variable and relatively small, it is difficult to attest the significance of this trend. However, we can make the following hypothesis: as software tools and reference databases have improved, the interpretation of WES variants has become less and less challenging for technology users. On the other hand, the publication of recommendations and guidelines in the USA [16], Canada [23] and Europe [22] has polarized the debate on the challenge of identification, classification and reporting of IF, which may help explain why it was increasingly mentioned in our selection of articles.

### Cost and reimbursement

The challenge of WES’ cost and of test reimbursement is reported in 49 % of articles (72). It includes a number of sub-challenges along the WES data trajectory from production to analysis and interpretation. Although they do not provide much detail, a number of articles published in 2014 and 2015 consider that WES is still too expensive to be implemented as a standard of care in different contexts such as epilepsy [54], acute myeloid leukemia [52], axonopathies [79], sudden unexplained death [80] and cardiac arrhythmia [78]. Since sequencing costs have fallen drastically over time, it is notable that even in 2014 some authors consider that it is the sequencing itself that is too expensive [37, 81–83]. Other justifications for the high price of these tests mentioned by authors include the cost of data storage [84] and necessary Sanger validation of WES results [50]. Data interpretation in general is another reason provided to explain the higher costs of WES compared to more targeted sequencing [50, 85, 86]. Focusing on the possibility of using WES in newborn screening, Beckmann et al. [87], provide a more detailed assessment of costs which leads them to conclude that “From a cost perspective, generalization of this practice with current procedures would entail a monumental effort that is likely to ruin our social healthcare programs.” Those “important social, economic, and human costs” are linked to the increased time clinicians would have to spend interpreting and reporting WES results to patients and families.

The large-scale application of WES in the clinic will only be possible if it is integrated fully into the healthcare system as a standard of care for certain conditions. This requires a thorough economic evaluation of possible funding sources and strategies to reimburse this sort of analysis. According to many articles from the USA, UK and Germany published after 2014, cost assessment analysis and economic evaluation studies still have to be performed in order to formally establish the relative cost efficiency of WES compared to other techniques [34, 38, 65, 73, 75, 88, 89]. The need for private insurance providers to reimburse these tests is reported as one of the key elements standing in the way of clinical implementation of WES on a larger scale, especially in the USA [39, 51, 89–91]. Not only will the clinical utility [62] and cost efficiency of these tests have to be proven, but insurance companies and the public healthcare system will have to organise the administrative infrastructure needed to reimburse those tests, such as by creating ‘new billing codes’ [39, 92].

## Discussion

Our methodology carried a number of limitations. The first challenge of our approach is that we tried to identify elements in publications which had a different primary focus. Indeed, we were looking to identify sections describing unsolved implementation challenges in publications focusing on the description of the actual use of WES in a clinical context. This made the task of identifying those sections more difficult, and may have resulted in failure to identify a number of articles. Indeed, the relevant sections of the selected articles were extremely diverse, ranging from a few words to full titled sections. Another issue which could possibly have led us to miss relevant publications was our choice of search terms. Our keyword combination of (“Clinical application” OR “Medical application” OR “Healthcare” OR “Clinical care” OR “Medical care” OR “Clinical practice” OR “Clinical diagnostic” OR “Medical practice”) may have lacked specificity, leading us to overlook relevant articles because of the very high number of hits we obtained. In addition, the process of filtering all 2095 articles was very lengthy. Since the date at which we performed the search, a number of potentially relevant articles have been published. In addition, the regulatory landscape of clinical WES has evolved, with the publication of a number of guidelines and recommendations which will significantly impact this field, notably in Europe [93], and the USA [94–97]. The speed, efficiency and reproducibility of the data filtering process could be significantly enhanced if this process was partly automated. However, to our knowledge there is no open access software tool that could have performed the search based on keywords and context generation more efficiently than we did. One other limitation lies in the combination of quantitative and qualitative approaches we used to analyze all 23 challenges identified in 147 selected articles. This was a relatively small sample size in which to obtain significant differences between sub-groups of articles. On the other hand, it was a high number of articles to analyze thoroughly, which is why we decided to analyze only the challenges that were most reported by authors. We believe this combination of qualitative and quantitative methodologies is key to making informed policy decisions based on the latest body of evidence regarding technologies such as WES.

## Conclusions

A number of challenges need to be resolved before whole exome sequencing can be implemented as a standard of care in the clinical setting. Through this systematic review of the literature, we could identify as many as 23 of these challenges. The three challenges that were most consistently reported by technology users were that of incidental findings, variants of unknown significance, and the cost of the technology. Although a small number of challenges, notably communication with patients, education of clinicians, and patients’ turnaround time, were reported differently in articles focusing on cancer, rare diseases or all diseases, and in articles from different countries, most challenges were discussed similarly across diseases and countries (data not shown). WES is already used in the clinical setting, and may soon be considered the standard of care for specific medical conditions, most notably for the identification of mutations contributing to rare genetic diseases. Clinics in the USA [41], France [98] and the Netherlands [99] already report promising results from the systematic use of NGS in hundreds of patients. Yet, technology users are calling for certain standards and guidelines to be published before this technology replaces more focused approaches such as gene panels sequencing. In addition, it is clear that a number of infrastructural adjustments will have to be made for clinics to store, process and analyze the amounts of data produced by WES. The interpretation of this data requires specially trained staff, and patients and families must also be adequately prepared to deal with WES test results. Some intermediary solutions may be found, such as the one suggested by Topper et al.: “In the near term, we suggest that many of these technical and ethical challenges may be alleviated by a targeted analysis approach, in which the full exome sequence is generated in patients, but analysis is initially limited to those genes already known to play a role in the presenting disorder” [60].

## Abbreviations

ACMG, The American College of Medical Genetics and Genomics; IF, incidental findings; NGS, next-generation sequencing; VUS, variants of unknown significance; WES, whole-exome sequencing; WGS, whole-genome sequencing

## Additional file

Additional file 1: PRISMA flow diagram. (DOCX 70 kb) Additional file 2: PRISMA checklist. (DOCX 28 kb) Additional file 3: Full articles dataset. (XLSX 628 kb)
