# Supplementary material for: Unsolved challenges of clinical whole-exome sequencing: a systematic literature review of end-users’ views
Source: BMC Med Genomics. 2016 Aug 11;9:52. doi: 10.1186/s12920-016-0213-6 (PMC4982236; doi:10.1186/s12920-016-0213-6)
Supplement: Additional file 1: — PRISMA flow diagram. (DOCX 70 kb) [file 12920_2016_213_MOESM1_ESM.docx]

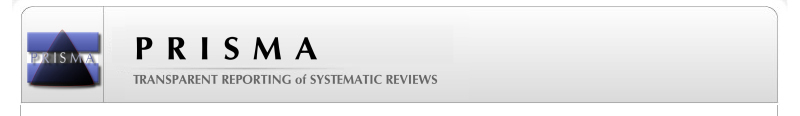
**PRISMA 2009 Flow Diagram**

Records excluded:

Not peer-reviewed journal articles written in English, Spanish or French
(n =302)

Full-text articles excluded: not written by a technology user, do not address whole-exome sequencing, do not discuss its clinical implementation do not list unsolved challenges
(n =1645)

Studies included in quantitative synthesis (meta-analysis)
(n =147)

Studies included in qualitative synthesis
(n =147)

Full-text articles assessed for eligibility
(n =1792)

Records screened
(n =2094)

Records after duplicates removed
(n =2094)

Additional records identified through other sources
(n = 0 )

## Identification

## Eligibility

## Included

## Screening

Records identified through database searching
(n = 2094)
